# Levels and Correlates of Physical Activity in Rural Ingwavuma Community, uMkhanyakude District, KwaZulu-Natal, South Africa

**DOI:** 10.3390/ijerph17186739

**Published:** 2020-09-16

**Authors:** Herbert Chikafu, Moses J. Chimbari

**Affiliations:** School of Nursing and Public Health, College of Health Sciences, University of KwaZulu-Natal, Durban 4001, South Africa; chimbari@ukzn.ac.za

**Keywords:** cardiovascular disease, healthy ageing, Ingwavuma, KwaZulu-Natal, modifiable behaviour, physical activity, rural, South Africa

## Abstract

Physical activity, among others, confers cardiovascular, mental, and skeletal health benefits to people of all age-groups and health states. It reduces the risks associated with cardiovascular disease and therefore, could be useful in rural South Africa where cardiovascular disease (CVD) burden is increasing. The objective of this study was to examine levels and correlates of physical activity among adults in the Ingwavuma community in KwaZulu-Natal (KZN). Self-reported data on physical activity from 392 consenting adults (female, *n* = 265; male, *n* = 127) was used. We used the one-sample *t*-test to assess the level of physical activity and a two-level multiple linear regression to investigate the relationship between total physical activity (TPA) and independent predictors. The weekly number of minutes spent on all physical activities by members of the Ingwavuma community was 912.2; standard deviation (SD) (870.5), with males having 37% higher physical activity (1210.6 min, SD = 994.2) than females (769.2, SD = 766.3). Livelihood activities constituted 65% of TPA, and sport and recreation contributed 10%. Participants without formal education (20%), those underweight (27%), and the obese (16%) had low physical activity. Notwithstanding this, in general, the Ingwavuma community significantly exceeded the recommended weekly time on physical activity with a mean difference of 762.1 (675.8–848.6) minutes, t (391) = 17.335, *p* < 0.001. Gender and age were significant predictors of TPA in level 1 of the multiple regression. Males were significantly more active than females by 455.4 min (*β* = −0.25, *p* < 0.001) and participants of at least 60 years were significantly less active than 18–29-year-olds by 276.2 min (*β* = −0.12, *p* < 0.05). Gender, marital status, and health awareness were significant predictors in the full model that included education level, employment status, body mass index (BMI), and physical activity related to health awareness as predictors. The high prevalence of insufficient physical activity in some vulnerable groups, notably the elderly and obese, and the general poor participation in sport and recreation activities are worrisome. Hence we recommend health education interventions to increase awareness of and reshape sociocultural constructs that hinder participation in leisure activities. It is important to promote physical activity as a preventive health intervention and complement the pharmacological treatment of CVDs in rural South Africa. Physical activity interventions for all sociodemographic groups have potential economic gains through a reduction in costs related to the treatment of chronic CVD.

## 1. Introduction

The burden of noncommunicable diseases, particularly cardiovascular disease (CVD), is increasing in low-to-medium income countries [1,2], South Africa included [3,4]. The rising burden of CVD in low- and middle-income countries (LMICs) is ascribed to multiple factors, including rising life expectancy, nutritional transition, poverty, improvement in income levels, and globalisation [5]. In addition to an age-related risk, unhealthy lifestyle practices exacerbate the risk of hypertension, type 2 diabetes mellitus (T2DM), and obesity that are part of CVD aetiology. Alcohol abuse, smoking, unhealthy diet, and physical inactivity also increase the risk of CVD [6,7].

However, health behaviour modification moderates the risk and onset of CVD. Physical activity confers health gains such as enhanced lipoprotein profile that reduces the likelihood of developing coronary heart disease (CHD) [8,9]. While any amount of physical activity, including walking, confers health benefits [10], sufficient moderate-to-vigorous-intensity physical activity augments the management of existing CVD conditions, enhances endothelial function, and lowers the of risk of atherosclerosis, hypertension, obesity, and T2DM [8]. In South Africa, a significant proportion of ischemic heart disease (IHD), ischemic stroke, T2DM, and breast cancer has been associated with physical inactivity [7].

Other than CVD, inactivity is related to numerous adverse outcomes. It is considered a risk factor for mental health disorders, chronic obstructive pulmonary diseases, some cancers, osteoporosis, and all-cause mortality [11,12,13,14]. The World Health Organization (WHO) recommends adults to perform at least 150 min of moderate-intensity physical activity or 75 min of vigorous-intensity physical activity or their combined equivalent across five days per week to moderate some of the risks [15]. The guidelines also recommend increasing physical activity to an equivalent of 300 min of moderate-intensity physical activity for additional health benefits. The health benefits of physical activity accrue across sociodemographic categories and health states, albeit relative to the volume of physical activity up to an optimum point [8,16,17].

It is essential to understand the levels and correlates of physical activity in rural South Africa for evidence-based public health interventions against the backdrop of the rising burden of noncommunicable diseases (NCDs) and ageing. Studies conducted in rural South Africa have shown significant inter and intraregional variation in physical activity levels across sociodemographic variables. Notably, physical activity mainly comprises of livelihood activities and varies with gender, age, marital status, and body mass index [18,19,20,21]. We, therefore, examined the levels and correlates of physical activity among adults aged 18 and above in Ingwavuma, a rural community in KwaZulu-Natal (KZN) addressing the following specific questions: (a) What is the level of physical activity in the community and does it meet WHO guidelines? (b) What are the components of physical activity in the community? And (c) What are the correlates of physical activity?

## 2. Methods

### 2.1. Study Design and Setting

This cross-sectional, observational and analytical study was conducted in Ingwavuma rural community (wards 13, 16, and 17) in Jozini Local Municipality under the uMkhanyakude district municipality in KZN, South Africa. Most households in the uMkhanyakude District depend on small scale subsistence agriculture [22] and social security disbursements for income [23,24]. The uMkhanyakude region experiences erratic rainfall ranging from 600 to 1000 mm per annum [25,26]. Fluctuant rainfall has affected forest cover and agricultural production. Consequently, poverty and food insecurity have worsened in uMkhanyakude [25,27], with only a quarter (26%) of households reporting sufficient food stocks at all times [23]. Jozini local municipality is the most populous municipality in uMkhanyakhude District and comprises of 20 administrative wards. It ranks among the least developed regions of the district with limited access to amenities including tapped water and has high levels of unemployment, poverty, and HIV/AIDS [24].

**Figure 1:**
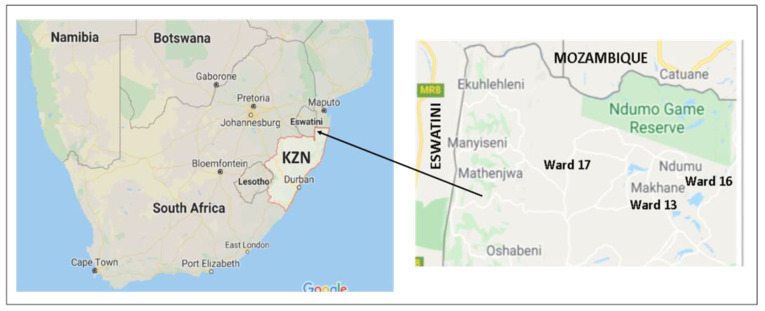
The map of South Africa showing Ingwavuma rural community in north-eastern KwaZulu-Natal (KZN). Source: Google Maps.

The Ingwavuma area is located in the north-eastern corner of KZN along the South Africa–Mozambique, and South Africa–Eswatini borders to the north and west, respectively (Figure 1). Like other rural areas in Jozini local municipality, Ingwavuma is inhabited by predominantly isiZulu speaking people under traditional leadership structures. The area is semi-arid and is characterised by three distinct seasons. It has a short winter season between June and August, followed by a hot, dry season until November and a humid summer season until March. The area is hilly with seasonal dams, streams, and rivers that sustain the community’s domestic water needs and small-scale gardening. Ingwavuma’s sociodemographic indicators are similar to other sub-regions in the district with high levels of poverty, unemployment, and HIV/AIDS. The area also has low levels of literacy and access to basic and social amenities.

### 2.2. Participants and Procedures

We enrolled adult (≥18 years) and non-disabled participants through convenience sampling from randomly selected villages in the study area. Data were collected over two phases. Firstly, the isiZulu questionnaire was administered to participants at their homes through face-to-face interviews by a team of three community research assistants (CRAs) supervised by the investigator. A trained nurse then conducted physical measurements. The CRAs were native isiZulu speakers and had completed at least high school (Metric Level for South Africa). Data were collected electronically using the KoBo-Collect application (Cambridge, MA, USA) on Android mobile devices [28]. This study was approved by the University of KwaZulu-Natal Biomedical Research Ethics Committee (BREC/00000235/2019), and all participants provided written informed consent.

### 2.3. Measures

We adapted the validated WHO STEPwise approach to surveillance (STEPS) questionnaire [29] to collect data on physical activity. The adaptation of the questionnaire was intended to contextualise questions and examples. The English version questionnaire was translated into isiZulu by a native speaker, revised for linguistic appropriateness with native isiZulu speaking community research assistants (CRAs) during training, and back-translated to English to ensure comparability with initial questions. We pretested the questionnaire in a village within the study area with 30 randomly selected and representative participants. The village was excluded from the main study. Minor revision of terminology and sequencing of questions was done using insights from the pretest. The final questionnaire with open-ended and closed-ended questions had the following five sections: sociodemographic data (age, gender, marital status, formal education level, and occupation), health awareness and knowledge, healthcare utilisation, and modifiable health behaviours.

The following self-reported physical activity data were collected: (a) forms of physical activity (livelihood, travel, and sport and recreational activities); (b) intensity level, whether moderate-intensity physical activity or vigorous physical activity; (c) weekly frequency; (d) estimate of time spent on physical activity; and (e) physical activity-related health awareness and knowledge. Time spent on physical activity was defined in moderate-intensity minutes, where a unit of vigorous-intensity equalled two units of moderate-intensity physical activity. Total physical activity (TPA) was the sum of the product of mean daily duration and weekly frequency of physical activity. The outcome variable TPA was expressed as a: (a) continuous variable to assess the level and predictors of physical activity, and (b) categorical variable to classify TPA against WHO recommendations.

The following categorical predictors were used in the analysis: Age (<30, 30–39, 40–49, 50–59, 60+ years), gender (male, female), formal education level (none, primary, secondary, post-secondary), employment status (unemployed, self-employed, employed, other), and body mass index (BMI) (underweight, normal-weight, overweight, obese). Two health-related predictors were also included, whether participants thought inactivity has adverse effects on health (yes, no, do not know) and whether participants were ever informed to be active (yes, no, do not remember).

### 2.4. Analyses

Descriptive statistics are presented as means (M) with their standard deviation (SD) for continuous variables, and as frequencies with corresponding percentages for categorical variables. We conducted the one-sample *t*-test to assess the community’s physical activity time against the recommended 150 min, and a two-level multiple linear regression to investigate the relationship between TPA and independent predictors. Model 1 controlled for age and gender as non-modifiable risk factors of physical activity. Formal education level, employment status, BMI, and proxies of health awareness were added into model 2 as modifiable factors. Non-modifiable factors are acquiescent to interventions [30], hence the use of hierarchical regression to study their influence on physical activity. We evaluated linear regression assumptions. Residual scatter plots of standardised predicted values were used to assess homoscedasticity and linearity, and both assumptions were met. Although visual inspection of histograms and residual plots did not suggest non-normal distribution, results of the Shapiro–Wilk test (SW) indicated a violation of the normality assumption (SW = 0.83, df = 392, *p* < 0.05). Nonetheless, multiple linear regression was used in view of the robustness of estimates from large sample data [31,32,33]. Statistical significance was evaluated at the 0.05 alpha level for all tests. We used IBM SPSS Statistics version 26 (IBM Corp., Armonk, NY, USA) to conduct statistical analyses.

## 3. Results

Table 1 presents an overview of participant characteristics. A total of 400 consenting participants were recruited into the study, and 392 participants with a mean age of 42.7 years (SD = 17.38) had complete data required for this assessment. The gender distribution was 67% (*n* = 265) female and 32% (*n* = 127) male. Participants in the 18–29 age group (*n* = 116, 30%) and 30–39 (*n* = 81, 21 %) constituted 50% of the sample. About two-thirds of the sample (*n* = 244, 62%) were single. The majority (*n* = 327, 83%) of study participants were unemployed with comparable levels across gender. The mean weight of the participants was 69.1 kg (SD = 18.2). One-fifth were obese, with a notably higher proportion of females (26%) than males (9%).

Results of the one-sample *t*-test show that the Ingwavuma community’s mean TPA (M = 912.2, SD = 870.5) significantly exceeds the WHO recommended 150 min of weekly physical activity, with a statistically significant mean difference of 762.1 (675.8–848.6), *t* (391) = 17.335, *p* < 0.001. Overall, the Ingwavuma community spent 912.2 (SD = 870.5) minutes on all physical activity (see supplementary file Table S1). However, there were distinct gender differences; males had 37% higher physical activity (1210.6 min, SD = 994.2) than females (769.2, SD = 766.3). Physical activity was also lower among participants aged at least 60 years (M = 714.5, SD = 721.9) and obese participants (M = 657.5, SD = 582.8) who were 33% less active than their normal-weight counterparts. Overall, livelihood activities contributed the most (65%) to TPA with almost even distribution between vigorous- and moderate-intensity activities. Two-thirds (67%) of participants indicated they grew crops in their fields to supplement their food requirements. On the other hand, sport and recreation had the least contribution (10%) to TPA. Time spent on all physical activity was consistently higher among males than females with a six-fold difference between the two genders in sport and recreational activities. Although there was no distinct age-related pattern on livelihood and travel physical activity, there were noteworthy differences in TPA in other predictors. Unmarried participants were most active, while the unemployed and obese ranked lowest. Time on sport and recreation varied inversely with age. Young adults under 30 years spent 201.6 min (SD = 306.9) on sport and recreational activities, while the elderly (60+ years) did not participate in similar activities. Additionally, only a fifth (23%) of participants believed that inactivity could result in adverse health outcomes.

**Figure 2:**
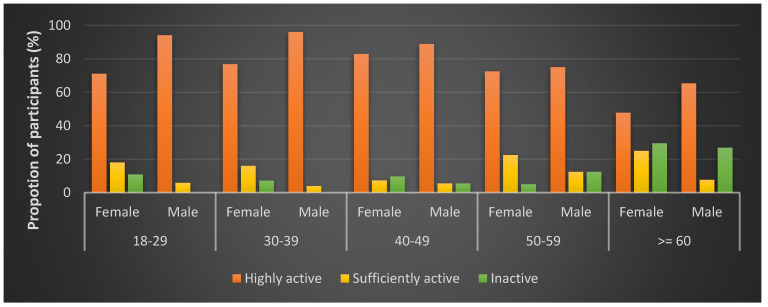
Physical activity classification by age and gender.

Table 2 summarizes the classification of physical activities. Almost a tenth (11%) and three quarters (75%) of study participants had low and high TPA levels, respectively. More males (84%) than females (70%) achieved high TPA, and most age groups had comparable proportions of participants in the three TPA classifications except for the 60+ year age group. Two-thirds of the elderly (60+ years) had low TPA, almost three-fold (28%) of the community level. Relative to other sub-categories, low physical activity was higher among participants without formal education (20%), the underweight (27%), and obese (16%). Adjusted for age, the prevalence of low TPA was more prevalent among females for all but those aged 50–59 (Figure 2). Insufficient activity levels also varied with gender. It was higher among females except for 45–59-year-olds and least among males aged 18–29 years (3%), 30–44-year-old males (0%), and females in the 45–59-year-old group (4%).

Results of the two-step hierarchical linear regression analysis conducted to understand sociodemographic predictors of TPA are presented in Table 3. From the set of dummy predictors entered into model 1 (gender and age group), the model significantly predicted TPA, F (5, 386) = 7.853, *p* < 0.001. A highly significant difference in TPA was predicted between males and females, with males significantly more active by 455.4 min (*β* = −0.25, *p* < 0.001). Compared with younger adults (<30 years), elderly participants aged at least 60 years (*β* = −0.12, *p* < 0.05) were predicted to attain significantly lower TPA. In the full model, male gender remained a significant predictor of higher TPA (*β* = 0.22, *p* < 0.05). From the set of modifiable factors added into model 2, being married (*β* = −0.12, *p* < 0.05), cohabiting (*β* = −0.13, *p* < 0.05), and lack of awareness on the adverse effects of inactivity were (*β* = −0.13, *p* < 0.05) were significant predictors of low TPA. Independent variables entered in model 2 significantly predicted the variability in TPA, F (20, 271) = 3.093, *p* < 0.001

## 4. Discussion

This study aimed to assess the levels and correlates of physical activity in rural KZN against the backdrop of the rising burden of CVDs and a trend towards sedentary living in South Africa. To the best of our knowledge, this study is the first to examine physical activity from a large sample of adults in the uMkhanyakude District of KwaZulu-Natal.

Participation in sports and recreational activities was low, with males participating more than females. This finding is consistent with evidence from studies in rural South Africa [34] and other African countries [35]. Low and gendered participation rate in sport and recreation is a concern amidst the rising prevalence of CVDs in rural South Africa. The low participation could be attributed to multiple factors, including a lack of awareness on the significance of sporting and recreational activities, unavailability of enabling infrastructure, and time constraints. The limited access to basic amenities in the area, notably tapped water, may result in time constraints for leisure activities. With such limitations, gendered roles and responsibilities from deep-rooted traditional practices [24] in the area are likely to affect women’s participation in leisure activities.

Although physical activity is known to be inversely related to age, the high prevalence of insufficient physical activity among the elderly is worrisome relative to age-related health risks. Substantive evidence shows ageing-induced adverse outcomes, such as elevated CVD risk, reduced skeletal muscle performance, increased risk of falls and loss of bone mass [36,37], and depression [38], can be moderated by physical activity. Physical activity also has an established positive effect on the mental health of the elderly [38].

Several factors potentially explain why the elderly in Ingwavuma are not leveraging the benefits of physical activity. First, the lack of awareness proxied by the high proportion of inactive participants unaware of the adverse health outcomes of inactivity potentially explains the trend among adults. Second, strenuous livelihood and household activities are associated with severe musculoskeletal disorders. The high prevalence of back pain and knee osteoarthritis among the elderly in rural South Africa [39,40] possibly explains the insufficient physical activity. Third, unlike typical rural areas dependent on labour-intensive subsistence farming, agricultural activities have declined in the region over the past few decades due to erratic rainfall [27]. Fourth, the high unemployment in the region [24] likely spares the elderly from livelihood activities and domestic chores as younger adults may assume a significant share of activities. In the absence of leisure activity, this results in physical activity deficits for the elderly.

Health education may help with awareness of the significance and spectrum of sport and recreation activities that do not require extensive hard infrastructure. Our findings indicate the need for health education to showcase the range of minimal cost physical activities services available to rural residents and their potential benefits. Aerobic activities with short bouts of vigorous activities (such as brisk walking) confer significant health benefits for all age-groups and health states [41,42,43]. Notwithstanding the risk of arrhythmias in older people during physical activity, light intensity activities such as regular walking have been established to be beneficial [10]. As such, the elderly in rural communities should be encouraged to incorporate walking into their leisure activity package and consider it as a health routine than exclusively as part of achieving livelihood and social activities. Importantly, health promotion will also help to influence social constructs that may hinder participation in sport and recreational activities.

Most physical activity in rural South Africa is associated with livelihood activities and is seasonal. Therefore, it is crucial to encourage sport and recreation in rural areas to assist with smoothing seasonal variation in physical activity. It is noteworthy that there was almost no report of sport and recreation among the elderly whose age predisposes them to CVD. Evidence shows that sport and recreational activities have considerable mental, bone and cardiovascular health benefits for the elderly, more so for those with sedentary habits [44,45,46]. Importantly, leisure-time physical activity also ensures healthy ageing [46]. Gender-based peer activity groups could be explored as a means to promote sport and recreation among the elderly and females of all age groups in rural South Africa.

Our study reports a number of limitations. We only assessed the correlates of physical activity due to the cross-sectional study design hence we could not examine causal relationships that underlie physical activity in Ingwavuma. Furthermore, we relied on a self-reported assessment of physical activity and acknowledge that assessments could have understated or overstated duration, frequency, and intensity, and therefore the extent of physical activity [47]. Notably, estimates were prone to a floor effect as household chores may not have been fully captured in physical activity reports.

Notwithstanding the aforementioned limitations, the study also had noteworthy strengths. The inclusion of adults of both genders allowed for a multilevel assessment of the influence of modifiable factors on physical activity in a rural setting. Furthermore, the large sample size allowed for the assessment of key sociodemographic correlates of physical activity that could be generalised for the community.

## 5. Conclusions

In conclusion, low participation in sport and recreational activities and the high proportion of the elderly with insufficient activity are worrisome given the rising burden of CVDs. Furthermore, it is essential to encourage rural communities to increase physical activity, given its benefits of enhancing lipoprotein balance [8] and healthy ageing [46]. Physical activity interventions have potential economic gains by reducing the treatment costs of chronic morbidity that may result from the lower prevalence and better control of CVD and its risk factors.

Our policy related recommendation is the promotion of physical activity as a primary health intervention that complements pharmacological treatment of CVDs in rural South Africa, where the burden of lifestyle-related morbidity is rising. We also recommend the provision of rudimentary infrastructure in some areas with rugged terrain to promote physical activity by enhancing neighbourhood walkability [48]. For optimum outcomes, it is needful to develop multilevel physical activity interventions relevant to rural social context. Future research should aim to understand knowledge, attitudes, and perceptions of physical activity; and its social nuances in rural areas. Longitudinal studies with objectively measured physical activity are recommended to provide extant estimates of energy expenditure and assess the determinants of physical activity.

## Figures and Tables

**Table 1:** Demographic characteristics of the Participants.

Variables		Female		Male		Total	
		*n* $\left(\bar{X}\right)$	% (SD)	*n* $\left(\bar{X}\right)$	% (SD)	*n* $\left(\bar{X}\right)$	% (SD)
Age		42.1	17.5	43.9	17.2	42.7	17.4
Age group	18–29	82	30.9	34	26.8	116	29.6
	30–39	56	21.1	25	19.7	81	20.7
	40–49	41	15.5	18	14.2	59	15.1
	50–59	40	15.1	24	18.9	64	16.3
	60+	46	17.4	26	20.5	72	18.4
Marital status	Single	166	62.6	78	61.4	244	62.2
	Married	42	15.9	20	15.8	62	15.8
	Cohabiting	36	13.6	25	19.7	61	15.6
	Widowed/divorced	21	7.9	4	3.2	25	6.4
Education level	None	88	33.2	35	27.6	123	31.4
	Primary school	67	25.3	38	29.9	105	26.8
	Secondary school	106	40.0	46	36.2	152	38.8
	Post-secondary	4	1.51	8	6.3	12	3.1
Occupational status	Unemployed	229	86.4	98	77.2	327	83.4
	Self employed	19	7.2	9	7.1	28	7.1
	Employed	4	1.5	10	7.9	14	3.6
	Other	13	4.9	10	7.9	23	5.9
Body mass index	Underweight	14	5.3	12	9.5	26	6.6
	Normal-weight	91	34.2	75	59.1	166	42.2
	Overweight	87	32.7	26	205	113	28.8
	Obese	74	27.8	14	11.0	88	22.4
Grow crops in field	No	86	32.5	45	35.4	131	33.4
	Yes	179	67.6	82	64.6	261	66.6
Inactivity may lead to poor health outcomes	No	177	66.8	97	76.4	274	69.9
	Yes	67	25.3	22	17.3	89	22.7
	Don’t know	21	7.9	8	6.3	29	7.4
Advised by a health worker to be physically active	No	109	41.0	57	44.9	166	42.2
	Yes	124	46.6	56	44.1	180	45.8
	Don’t remember	32	12.4	14	11.0	46	12.0
**Total**		**265**	**100**	**127**	**100**	**392**	**100**

Notes: $\bar{X}$—mean; SD—Standard deviation.

**Table 2:** Classification of weekly physical activity.

Variable		Weekly Physical Activity Level (%)		
		Low	Sufficient	High
Overall		10.9	14.3	74.8
Gender	Female	12.0	17.7	70.3
	Male	8.7	7.1	84.3
Age group	<30	7.7	14.5	77.8
	30–39	4.9	12.4	82.7
	40–49	8.5	6.8	84.8
	50–59	7.8	18.8	73.4
	60+	27.8	18.1	54.2
Marital status	Single	6.5	12.2	81.2
	Cohabiting	16.1	19.4	64.5
	Married	14.8	16.4	68.9
	Other	32.0	16.0	52.0
Education	None	19.5	13.8	66.7
	Primary	6.7	19.1	74.3
	Secondary	7.2	12.4	80.4
	Post-secondary	8.3	0.0	91.7
Occupational status	Unemployed	12.5	14.0	73.5
	Self-employed	3.6	17.9	78.6
	Employed	0.0	7.1	92.9
	Other	4.4	17.4	78.3
Body mass index	Underweight	26.9	7.7	65.4
	Normal-weight	7.8	13.3	78.9
	Overweight	8.6	12.9	78.5
	Obese	16.1	17.3	66.7
Inactivity may lead to poor health outcomes	No	16.7	16.7	66.7
	Yes	8.4	13.1	78.5
	Don’t know	17.2	17.2	65.5
Advised to be physically active	No	10.8	17.5	71.7
	Yes	11.7	11.1	77.2
	Don’t remember	8.5	14.9	76.6

**Table 3:** Results of hierarchical linear regression on time spent on physical activity in Ingwavuma (*N* = 392).

Predictors		Model 1				Model 2			
		B	β	Sig.	95% CI	B	β	Sig.	95% CI
Constant		747.6		0.000	586.5–908.6	873.5		0.000	647.2–1099.7
Gender	(Female) Male	455.4	0.25	0.000	277.9–633.0	408.1	0.22	0.000	214.0–602.2
Age-group	(18–29) 30–39	164.4	0.08	0.175	−73.3–402.0	196.9	0.09	0.126	−55.8–449.6
	40–49	228.6	0.09	0.088	−33.8–491.1	293.7	0.12	0.067	−20.3–607.7
	50–59	−3.3	−0.00	0.980	−259.2–252.7	107.1	0.05	0.548	−243.4–457.7
	≥60	−276.2	−0.12	0.028	−522.8–−29.7	−109.5	−0.05	0.547	−467.2–248.1
Education	(Secondary) None					68.8	0.04	0.653	−231.8–369.4
	Primary					−37.0	−0.02	0.758	−280.0–204.1
	Post-secondary					−84.5	−0.02	0.744	−592.2–423.2
Marital status	(Single) Married					−259.1	−0.12	0.037	−503.0–−15.1
	Cohabiting					−322.3	−0.13	0.013	−577.4–−67.1
	Other					−313.2	−0.09	0.102	−688.6–62.2
Employment status	(Unemployed) Self employed					213.2	0.06	0.207	−118.6–545.1
	Employed					163.3	0.04	0.493	−304.7–631.3
	Other					−175.3	−0.05	0.362	−552.6–202.0
BMI	(Normal-weight) Underweight					−68.0	−0.02	0.704	−419.2–283.2
	Overweight					−6.5	−0.00	0.951	−215.8–202.8
	Obese					−203.7	−0.01	0.087	−437.4–29.9
Inactivity has adverse effects?	(Yes) No					−160.9	−0.08	0.156	−383.2–61.4
	Don’t know					−339.7	−0.10	0.040	−663.8–−15.5
Advised to be active	(No) Yes					42.2	0.03	0.530	−89.9–174.4
R-squared		0.92		0.000		0.14		0.118	
Adjusted- R-squared		0.81				0.10			

Note: Reference categories of predictors are given in brackets. B—Beta coefficient; CI—Confidence interval.

## Supplementary Materials

The following are available online at https://www.mdpi.com/1660-4601/17/18/6739/s1, Table S1: Mean weekly minutes (standard deviation; SD) spent on livelihood, travel, and sport and recreational activities in Ingwavuma.
