# Supplementary material for: Levels and Correlates of Physical Activity in Rural Ingwavuma Community, uMkhanyakude District, KwaZulu-Natal, South Africa
Source: Int J Environ Res Public Health. 2020 Sep 16;17(18):6739. doi: 10.3390/ijerph17186739 (PMC7559597; doi:10.3390/ijerph17186739)
Supplement: Supplementary file 1 [file ijerph-17-06739-s001.pdf]

Table S1. Mean weekly minutes (SD) spent on livelihood, travel and sport and recreational activities in Ingwavuma, N = 392

**Variable**		N	**Livelihood activities**		**Travel**	**Sport and recreation**	**Total**
			Moderate intensity	Vigorous intensity			
Overall		392	323.1 (440.2)	270.8 (489.3)	225.7 (285.0)	92.6 (244.9)	912.2 (870.5)
Gender	Female	265	311.6 (444.9)	231.8 (415.1)	191.2 (232.4)	34.6 (97.0)	769.2 (766.3)
	Male	127	347.2 (431.1)	352.2 (610.1)	297.6 (362.2)	213.5 (380.1)	1, 210.6 (994.2)
Age group	18-29	116	284.7 (345.7)	180.7 (386.5)	222.84 (283.5)	192.8 (293.0)	881.0 (756.8)
	30-39	81	399.4 (557.2)	295.6 (531.5)	238.0 (291.7)	119.4 (331.3)	1, 052.45 (998.4)
	40-49	59	394.1 (470.2)	398.2 (575.0)	267.0 (349.1)	55.9 (191.7)	1, 115.1 (900.5)
	50-59	64	318.1 (445.7)	381.9 (575.9)	200.1 (223.0)	15.0 (74.8)	915.1 (972.9)
	>= 60	72	245.4 (384.0)	185.1 (384.6)	205.3 (273.9)	0	635.8 (696.1)
Education level	None	123	320.9 (420.8)	290.2 (491.5)	213.7 (252.9)	10.2 (69.9)	835.1 (837.3)
	Primary	105	346.2 (536.7)	287.1 (536.1)	220.4 (298.5)	58.4 (161.3)	912.2 (916.3)
	Secondary	152	316.5 (392.8)	254.2 (469.4)	240.4 (305.0)	152.5 (301.5)	963.5 (881.2)
	Post-secondary	12	227.5 (259.2)	140.0 (248.8)	208.3 (231.2)	477.5 (533.7)	1, 053.3 (662.9)
Marital status	Single	244	333.1 (453.3)	311.5 (546.9)	246.7 (304.6)	128.1 (272.8)	1, 019.5 (938.8)
	Married	62	370.4 (496.2)	207.2 (344.5)	173.3 (200.6)	47.4 (239.5)	798.3 (750.4)
	Cohabiting	61	280.5 (375.1)	206.6 (383.5)	204.1 (292.9)	34.4 (129.8)	725.6 (684.3)
	Widowed/divorced	25	212.0 (273.7)	188.0 (398.5)	202.6 (232.9)	0	602.6 (693.7)
Occupational status	Unemployed	327	319.0 (441.7)	262.3 (450.7)	228.5 (291.3)	75.8 (237.6)	885.7 (855.4)
	Self employed	28	288.2 (378.1)	472.1 (864.8)	253.6 (318.7)	150.0 (320.3)	1, 164.1 (1095.0)
	Employed	14	572.1 (621.8)	240.0 (447.0)	226.8 (227.2)	258.2 (253.3)	1, 297.1 (857.1)
	Other	23	272.2 (325.1)	165.7 (381.4)	150.2 (156.4)	160.4 (185.5)	748.48 (712.8)
BMI	Underweight	26	363.9 (457.2)	267.3 (503.9	199.6 (284.7	113.1 (356.8)	943.9 (1, 051.6)
	Normal weight	166	361.5 (503.0)	298.4 (520.3)	241.9 (315.6)	129.3 (276.2)	1, 031.2 (918.3)
	Overweight	113	299.7 (381.9)	289.2 (519.8)	240.6 (281.7)	76.2 (229.1)	905.7 (827.84)
	Obese	88	268.4 (371.4)	196.4 (370.7)	183.7 (221.1)	38.2 (123.5)	686.6 (731.9)

Variable		N	Livelihood activities		Travel	Sport and recreation	Total
			Moderate intensity	Vigorous intensity			
Inactivity may lead to poor health outcomes	No	274	265.0 (374.2)	258.4 (474.0)	205.7 (241.2)	59.0 (241.9)	788.1 (863.1)
	Yes	89	356.6 (469.1)	284.4 (504.9)	241.3 (303.4)	103.7 (250.1)	986.0 (893.1)
	Don't remember	29	185.2 (280.8)	181.2 (376.2)	139.1 (206.9)	91.0 (196.2)	596.6 (508.9)
Advised to be physically active	No	166	295.8 (406.0)	313.3 (564.2)	222.3 (312.2)	81.6 (190.6)	912.9 (949.6)
	Yes	180	371.5 (494.5)	224.5 (411.0)	230.5 (273.5)	100.4 (263.8)	926.9 (830.5)
	Don't remember	46	233.6 (296.8)	299.4 (477.0)	218.9 (227.5)	101.5 (329.3)	853.4 (733.7)
